# Long-term persistence of knee pain and occupational exposure in two large prospective cohorts of workers

**DOI:** 10.1186/1471-2474-15-411

**Published:** 2014-12-05

**Authors:** Eléonore Herquelot, Julie Bodin, Audrey Petit, Catherine Ha, Annette Leclerc, Marcel Goldberg, Marie Zins, Yves Roquelaure, Alexis Descatha

**Affiliations:** UMS011 Inserm, Université Versailles St-Quentin, Population-Based Epidemiological Cohorts, Hôpital P. Brousse - bat 15/16 RDC Gauche, 16 av Paul Vaillant Couturier, 94807 Villejuif Cedex, France; LUNAM Université, Université d’Angers, Laboratoire d’ergonomie et d’épidémiologie en santé au travail (LEEST), Angers, France; Institut de veille sanitaire (InVS), Département santé travail, Saint-Maurice, France; AP-HP, Poincaré University Hospital, Occupational Health Unit, Garches, France

**Keywords:** Musculoskeletal pain, Occupational exposure, Persistence

## Abstract

**Background:**

The persistence of knee pain (KP) and its relationship with occupational factors were investigated in two prospective cohorts of French workers: retirees of the Gazel cohort and workers in the Cosali cohort.

**Methods:**

KP was defined according to the Nordic questionnaire (>1 day in the last year), and the information was extracted from two questionnaires in 2006 and 2012 for the Gazel cohort, and in 2002–2005 and 2007–2010 for the Cosali cohort. The personal and occupational factors and the severity of KP were measured at baseline. Of the 4590 members of the Gazel cohort with KP at baseline, 4140(90.2%) were followed up, as were 637(63.1%) members of the Cosali cohort. Logistic models were used to evaluate associations (ORs) between occupational exposure and the persistence of KP separately by sex, adjusted on indicators of severity of KP.

**Results:**

KP was no longer present at follow-up for 38.3% of Gazel men and 46.0% of Cosali men (33.4% of Gazel women and 50.6% of Cosali women). The persistence of KP in men was associated with carrying or handling heavy loads on univariate analyses and with kneeling on multivariate analyses, with ORs of 1.3(1.0-1.6) (Gazel) and 1.6(1.0-2.6) (Cosali). Climbing stairs was not significantly associated with the persistence of knee pain among men. The persistence of KP in women was not significantly associated with such occupational exposure.

**Conclusions:**

This study highlights the role of occupational factors in the persistence of KP for men, in particular kneeling and handling/carrying loads.

**Electronic supplementary material:**

The online version of this article (doi:10.1186/1471-2474-15-411) contains supplementary material, which is available to authorized users.

## Background

Knee pain (KP) is a common condition in the adult population, and a recent German study reported a prevalence of 31% in the general population aged over 40 years [1]. The annual recovery rate from KP has been estimated to be between 8.7 and 51.0%, depending on the definition of recovery and the populations considered [2–7].

The natural history of KP has mainly been studied according to clinical criteria or to certain individual and psychological factors [8]. In terms of clinical factors the persistence of KP has been found to be associated with previous episodes of pain, pain in other locations and high intensity of pain [2, 3]. In terms of individual factors, age, a high BMI and being female were associated with persistence of KP; and the psychological factors were poor mental health and living alone [3].

Recent reviews have highlighted a relationship between the prevalence and the incidence of KP or knee osteoarthritis and occupational factors, generally physical workload and more particularly kneeling and lifting weights [9]. Occupational exposure has been shown to be associated with the severity and duration of episodes of KP [10] and may lead to persistence of KP. The influence of occupational factors on the persistence of KP has been rarely studied. One study involving workers in the forestry industry found that low job satisfaction and repetitive twisting movements of the trunk were associated with the persistence of KP [5]. Another study among female healthcare workers did not find any association between perceived exertion and the one-year persistence of KP [6], and another study found higher persistence of KP for bricklayers than for construction supervisors [7].

The relationship between occupational factors and the persistence of KP needs to be explored further. We therefore decided to assess the recovery rate from KP and the occupational risk factors for the persistence of KP in two large prospective cohorts of workers, i.e. the Gazel cohort and the Cosali cohort.

## Methods

### The Gazel cohort

**Figure 1:**
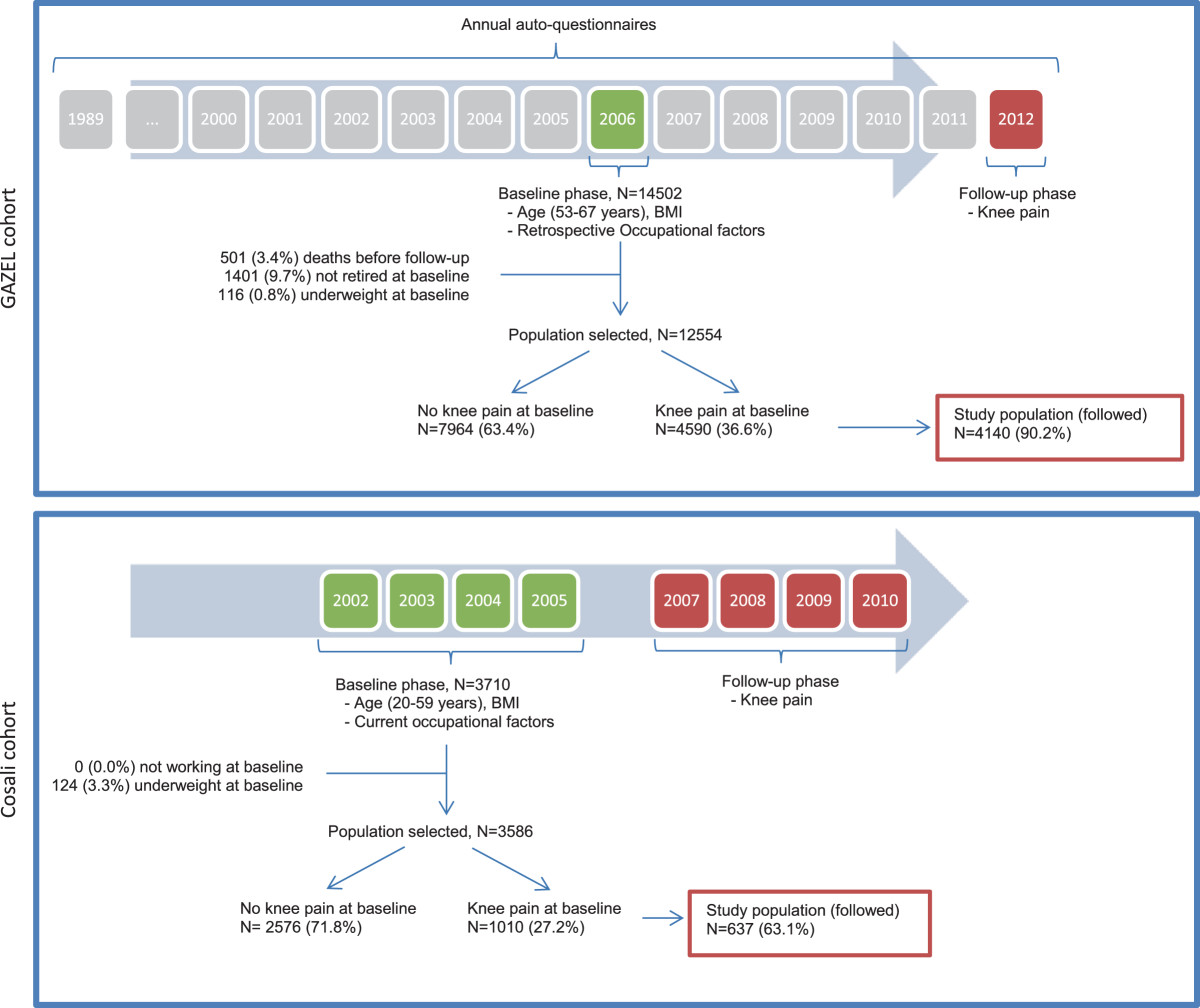
**Structure of Gazel and Cosali cohorts.**

The GAZEL cohort was established in 1989 from the employees of Electricité de France (EDF) and Gaz de France (GDF), the French national utility for energy production and distribution. The company employs approximately 150,000 people of various trades and socioeconomic status throughout France, and EDF-GDF employees hold civil servant–like status that entails job security. At baseline in 1989, the cohort included 20,625 volunteers (men aged 40 to 50 years and women aged 35 to 50 years). In the January of each successive year, participants received a general questionnaire about their lifestyle, health, and occupational situation [11]. Medically-certified sickness absence data were available from company records.In this study, we focused on the 14,502 participants who had responded to the 2006 questionnaire (Figure 1). The questionnaires sent in 2006 and 2012 contained questions concerning the information of interest in this study: in the following text, 2006 is called the “baseline” phase and 2012 is called the “follow-up” phase.

### The Cosali cohort

This prospective study was based on two successive surveys in a large sample of workers in the Loire Valley area of West Central France, the Cosali cohort [*Cohorte des Salariés Ligériens*[12]]. The diversity of the regional economic structure (5.6% of the French workforce) is very similar to that of the national workforce [13].

At the time of the first survey, all French employees, including temporary and part-time workers, were required to undergo an annual health examination by an occupational physician (OP) in charge of the medical surveillance of a group of companies. The 83 OPs who volunteered for the study (without compensation) randomly selected workers aged between 20 and 59 years from those undergoing one of these annual health examinations between 2002 and 2005. Subjects filled in self-administered questionnaires before the OP’s physical examination [Surveillance network of musculoskeletal disorders [14]]. This first phase is called the “baseline” phase in the following text.

A follow-up questionnaire was sent to the 3,710 participants in 2007 [12] (Figure 1). In the case of non-response, two successive reminder letters were sent. This second phase is called the “follow-up” phase in the following text.

### Study population

Workers who were underweight (BMI < 18.5 kg/m^2^) were excluded from the analyses in this study because this may correspond to a variety of other medical conditions [15].

In order to have homogeneous measures of occupational exposure, members of the Gazel cohort who were not retired at baseline or who were deceased before the follow-up phase were excluded. The members of the Cosali cohort were all active workers at baseline.Workers who were not followed up were excluded from the analyses in both cohorts (Figure 1).

### Outcome

Participants completed the standardized Nordic questionnaire for several musculoskeletal symptoms including KP [16]. KP was defined as at least 1 day of KP during the preceding year. Persistent cases were those who reported KP both in the baseline phase and in the follow-up phase. The KP was described according to pain duration and period in two classes: long-lasting pain (>30 days during the preceding year) and other pain (1 to 30 days during the preceding year or only during the preceding week).

### Risk factors

#### Personal factors

Age at the time of the baseline questionnaire was divided into 5 classes: 20–39 years, 40–49 years, 50–59 years, 60–64 years and 65–69 years.

Weight and height were self-reported in the baseline questionnaire in the both cohorts. The Body Mass Index (BMI) at baseline was divided into three classes: normal weight/missing (18.5 to 25 kg/m^2^ or missing), overweight (25 to 30 kg/m^2^) and obese (>30 kg/m^2^).

The Gazel cohort members were asked about their history of knee injury before 2006 (Yes/No). Sickness absence for depression during the whole career (ever/never) was taken into account, since pain may be associated with depression.

#### Characteristics of KP at baseline

The intensity of KP from the Nordic questionnaire at baseline was dichotomized into slight pain (1 to 4 on the 8-degree scale in Gazel, 1 to 5 on the 10-degree scale in Cosali) and severe pain (5 to 8 in Gazel, 6 to 10 in Cosali). The Gazel cohort members had to describe the intensity of the pain in the last episode and the Cosali cohort members had to answer only if they had pain at the time of the questionnaire.

The baseline questionnaire also investigated nine other musculoskeletal symptoms (fingers, hands, elbows, shoulders, neck, upper back, lower back, hips and ankles). A composite variable called “Pain in other areas” focusing on pain in joints in the body other than the knees was divided into three classes: no pain in other areas, pain in hips (with or without other areas) and pain experienced in at least one of the eight other areas.

The Gazel cohort members were asked to self-assess the origin of KP with four pre-coded categories: tendinitis, meniscus disorders, arthrosis, other origin. For the analyses, three categories were considered: degenerative osteoarthritis, other origin (tendinitis, meniscus disorders or other), not completed.

#### Occupational factors

The socio-professional category at 35 years was available for all Gazel cohort members and at baseline for the Cosali cohort members. We divided this characteristic into 4 classes: manual workers, white collar, associate professionals/technicians and executives/others (craftsman, shopkeeper, business owner, managers, executives or not completed).

In the 2006 questionnaire, the Gazel cohort members reported the cumulative duration of exposure to three biomechanical constraints during their careers, i.e. carrying heavy loads (more than 10 kg), working in kneeling/stooping position and walking up more than 10 flights of stairs every day. Four answers were possible for each type of exposure: never, less than 10 years, 10–20 years, and longer than 20 years. We divided exposure into three classes: none (never or missing), short (less than 10 years) and long (more than 10 years).

In the 2002–2005 questionnaire, the Cosali cohort members reported current exposure during a typical working day to two types of biomechanical exposure: handling heavy loads (more than 4 kg) and working in a kneeling position. The response categories for occupational exposure were initially presented on a 4-point Likert-type scale as never or almost never, rarely (less than 2 hours a day), often (2 to 4 hours a day) and always (more than 4 hours a day). We divided the exposure categories into 3 classes: none (never or almost never or not completed), moderate (<2 hours a day) and severe (2 to 4 hours a day or more than 4 hours a day). Handling loads was also available at the follow-up questionnaire. The occupational changes since 2002 were reported at follow-up, in four categories (no change; change of job in the same company; change of company; not actively employed).

Length of exposure was also divided into short or long/moderate or severe exposure vs no exposure (none) in both cohorts in order to have sufficient statistical power in the analyses.

### Statistical analyses

All analyses were performed separately for each cohort and sex and the results are described separately.

The annual recovery rate from KP in each cohort was approximated by the rates of recovery from KP during the follow-up period multiplied by the estimated proportion of recoveries one year after baseline. This last figure was estimated from the percentage of Gazel members that did not declare shoulder/elbow/hip/knee pain in the annual questionnaires during the follow-up period. The natural history of KP was described according its duration at baseline and at follow-up. As the participants lost to follow-up were excluded from the analyses, a sensitivity analysis on the persistence of KP was performed assuming two extreme scenarios on the missing values: a pessimistic scenario, in which all missing individuals had KP at follow-up and an optimistic scenario, in which no missing individuals had KP at follow-up.

The main analyses consisted of evaluating associations between risk factors and the persistence of KP (i.e. reporting of KP in the baseline and the follow-up questionnaires). Univariate and multivariate logistic models were used to estimate the odds ratios (ORs) for persistence of KP. Wald tests were used to compare ORs between categories, in particular to test whether there were some dose-effect relationships for occupational exposure.

Two multivariate models were used to evaluate the relationship with carrying/handling heavy loads or kneeling with adjustment on:

- Age and BMI (called model 1)
- Age, BMI, intensity of pain, pain in other areas, self-assessed origin of KP (Gazel only), past history of knee injury (Gazel only) (called model 2)

The second-order interactions between the associations of KP with occupational factors and indicators of severity of KP were explored in model 2.

Data analyses for this study were generated with SAS 9.4 software (SAS Institute Inc., Cary, NC, USA). Statistical significance was defined as a p-value lower than 0.05.

### Ethics approval

Each cohort was approved by the French National Data Protection Committee (CNIL, Commission Nationale de l’Informatique et des Libertés) and all participants have given their consent to be entered in the cohorts.

## Results

### Study population

Of the 14,502 Gazel cohort members, 12,554(86.6%) met the inclusion criteria and 36.6% had KP at baseline (Figure 1). Of the 3,710 Cosali cohort members, 3,586(96.7%) met the inclusion criteria and 27.2% had KP at baseline. A follow-up questionnaire was available for 90.2% of Gazel cohort members (4,140 participants) and for 63.1% of Cosali cohort members (637 participants).

At baseline, KP had lasted more than 30 days for 43.5% of Gazel cohort members and for 34.2% of Cosali cohort members (Table 1). Around 90% of cases of KP at baseline (89.2% Gazel, 93.6% Cosali) had pain in other areas, including 26.2% with hip pain in the Gazel cohort and 34.1% in the Cosali cohort. In the Gazel cohort, 45.7% of cases of KP were reported as due to degenerative osteoarthritis and 37.8% of participants with KP declared a past history of knee injury.

**Table 1:** **Baseline characteristics of the study populations with knee pain at baseline**

	Gazel		Cosali	
	Men (N = 3190)	Women (N = 950)	Men (N = 356)	Women (N = 261)
Duration of follow-up, in years*	5.99 (5.98-6.00)	5.99 (5.98-6.00)	3.85 (3.11-4.86)	3.66 (2.87-4.83)
**Age, in years**				
20-39	-	-	159 (42.3%)	98 (37.5%)
40-49	-	-	132 (35.1%)	91 (34.9%)
50-59	709 (22.2%)	324 (34.1%)	85 (22.6%)	72 (27.6%)
60-64	1657 (51.9%)	420 (44.2%)	-	-
65-69	**824 (25.8%** **)**	**206 (21.7%** **)**	-	-
**BMI**				
Normal weight, missing	1074 (33.7%)	**485 (51.0%** **)**	**191 (50.8%** **)**	**159 (60.9%** **)**
Overweight	**1597 (50.1%** **)**	317 (33.4%)	146 (38.8%)	74 (28.3%)
Obesity	**519 (16.3%** **)**	**148 (15.6%** **)**	39 (10.4%)	28 (10.7%)
**Socio-professional category**				
Executives, others, missing	**541 (17.0%** **)**	111 (11.7%)	33 (8.8%)	12 (4.6%)
Associate professionals or technicians	**1834 (57.5%** **)**	**504 (53.0%** **)**	99 (26.3%)	44 (16.9%)
Employees	160 (5.0%)	330 (34.7%)	26 (6.9%)	**128 (49.0%** **)**
Manual workers	655 (20.5%)	5 (0.5%)	**218 (58.0%** **)**	77 (29.5%)
Carrying/Handling heavy loads**				
None	918 (28.8%)	**667 (70.2%** **)**	**162 (43.1%** **)**	149 (57.1%)
Short or moderate exposure	**938 (29.4%** **)**	99 (10.4%)	86 (22.9%)	47 (18.0%)
Long or severe exposure	**1016 (31.8%** **)**	29 (3.0%)	89 (23.7%)	27 (10.3%)
Missing	318 (10.0%)	155 (16.3%)	39 (10.4%)	38 (14.6%)
**Kneeling**				
None	1082 (33.9%)	**737 (77.6%** **)**	126 (33.5%)	105 (40.2%)
Short or moderate exposure	970 (30.4%)	40 (4.2%)	**132 (35.1%** **)**	**96 (36.8%** **)**
Long or severe exposure	779 (24.4%)	9 (0.9%)	**115 (30.6%** **)**	**59 (22.6%** **)**
Missing	359 (11.2%)	164 (17.3%)	3 (0.8%)	1 (0.4%)
**Occupational change between 2002 and follow-up**				
No change			206 (54.79%)	141 (54.02%)
Change of job in the same company			70 (18.62%)	47 (18.01%)
Change of company			45 (11.97%)	32 (12.26%)
Inactive/ missing			52 (14.63%)	41 (15.71%)
**Walking up more than 10 flights of stairs**				
None	1883 (59.0%)	750 (78.9%)		
Short exposure	551 (17.3%)	20 (2.1%)		
Long exposure	276 (8.6%)	8 (0.8%)		
Missing	480 (15.0%)	172 (18.1%)		
**Intensity of pain**				
Low, Missing	1741 (54.6%)	515 (54.2%)	106 (28.2%)	61 (23.4%)
High	1449 (45.4%)	435 (45.8%)	95 (25.3%)	64 (24.5%)
No actual pain	-	-	175 (46.5%)	136 (52.1%)
**Past history of knee injury**				
No, missing	1922 (60.3%)	652 (68.6%)		
Yes	1268 (39.7%)	298 (31.4%)		
**Self-assessed origin of knee pain**				
Degenerative osteoarthritis	1349 (42.3%)	543 (57.2%)		
Other	1559 (48.9%)	305 (32.1%)		
Not completed	282 (8.8%)	102 (10.7%)		
**Knee pain at baseline**				
Long-lasting pain	**1310 (41.1%** **)**	**490 (51.6%** **)**	124 (32.98%)	94 (36.02%)
Other pain	1880 (58.9%)	460 (48.4%)	**252 (67.02%** **)**	**167 (63.98%** **)**
**Pain in other areas at baseline**				
No pain	**380 (11.9%** **)**	**69 (7.3%** **)**	33 (8.8%)	8 (3.1%)
Hip pain	740 (23.2%)	345 (36.3%)	**110 (29.3%** **)**	**107 (41.0%** **)**
Pain in other areas	2070 (64.9%)	536 (56.4%)	233 (62.0%)	146 (55.9%)

*Median (q1 -q3).

**Gazel: Carrying loads of more than 10 kg, Cosali: Handling loads of more than 4 kg.

In bold: Significant difference between categories (p < 0.05)

In the Cosali cohort, 66.9% of male workers and 57.7% of female workers exposed to handling loads at baseline also reported being exposed at follow-up (results not shown). Around 54% of workers reported no occupational change between 2002 and the follow-up.

### Natural history of KP

**Figure 2:**
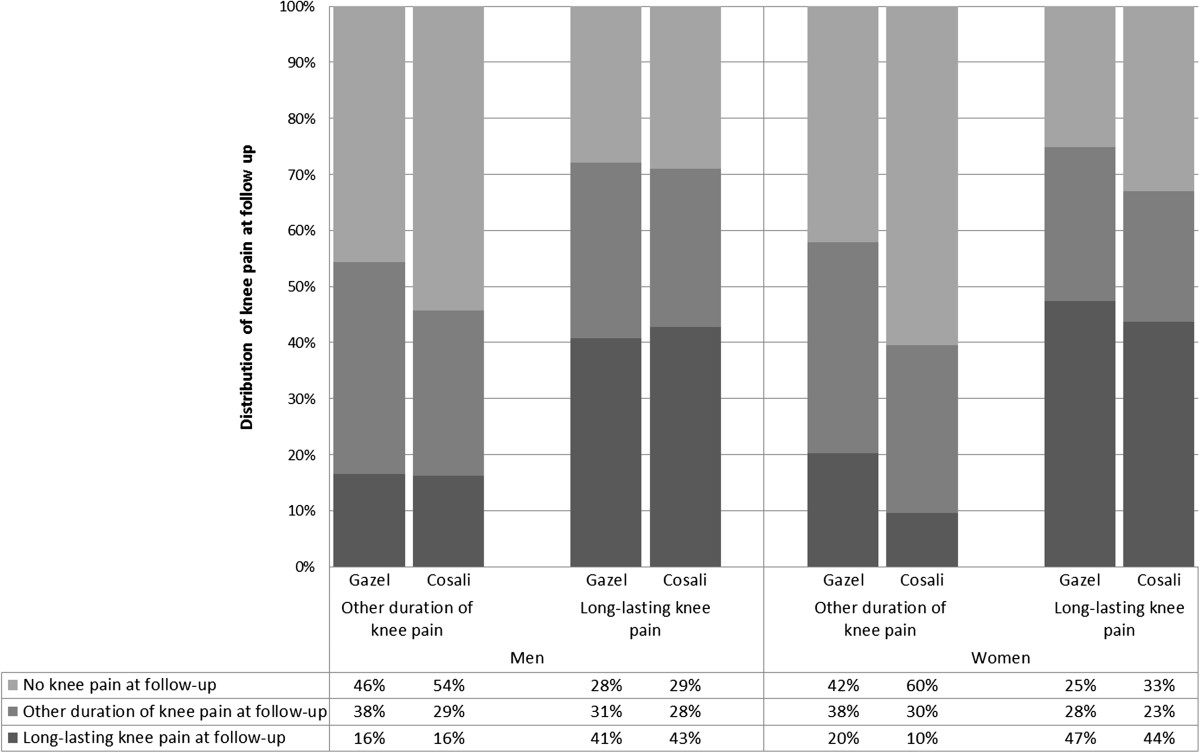
**Natural history of knee pain according the duration of pain at baseline and sex.**

KP was no longer present at follow-up for 38.3% of Gazel men and 46.0% of Cosali men. Knowing that 41.4% of recoveries seemed to occur before one year of follow-up in Gazel men, and thus the annual recovery rate from KP was estimated to be 15.9% for Gazel and 19.1% for Cosali. Of the men with long-lasting KP at baseline, from 27.9% to 29.0% did not report KP at follow-up, 40.7% to 42.7% had long-lasting KP at follow up and 28.2% to 31.4% had other durations of KP at follow-up (Figure 2). Among men with other durations of KP at baseline, from 45.6% to 54.4% did not report KP at follow-up, 16.3% to 16.5% had long-lasting KP at follow up and 29.4% to 37.9% had other durations of KP at follow-up.

KP was no longer present at follow-up for 33.4% of Gazel women and 50.6% of Cosali women. At least 35.8% of recoveries seemed to occur before one year of follow-up in Gazel women, and thus the annual recovery rate from KP was estimated at 11.9% for Gazel and at 18.0% for Cosali. Of the women with long-lasting KP at baseline, from 25.1% to 33.0% did not report KP at follow-up, 43.6% to 47.3% had long-lasting KP at follow-up and 23.4% to 27.5% had other durations of KP at follow-up. Among women with other durations of KP at baseline, from 42.2% to 60.5% did not report KP at follow-up, 9.6% to 20.2% had long-lasting KP at follow up and 29.9% to 37.6% had other durations of KP at follow-up.

The optimistic and the pessimistic scenarios indicated that the 34.9% to 43.9% of Gazel men and from 27.9% to 67.3% of Cosali men recovered from KP (and 29.2% to 41.7% of Gazel women and 33.8% to 66.9% of Cosali women).

### Occupational factors associated with persistence of KP

On univariate analyses, handling/carrying loads and kneeling for long durations were significantly associated with the persistence of KP for Gazel men, and dose–response effects were found with a higher risk of persistence for long exposure than for short exposure (p = 0.0006 for carrying/handling loads and p = 0.0052 for kneeling) (Table 2). More broadly, being exposed to these occupational factors (short and long exposure or moderate and severe exposure) was significantly associated with higher levels of persistence of KP for men in both cohorts (ORs around 1.5-1.6). Walking up more than 10 flights of stairs was not associated with the persistence of KP. Some socio-professional categories (i.e. executives compared to other categories) were associated with lower persistence in Cosali men. The persistence of KP in women was not found to be significantly associated with occupational exposures in either Gazel or Cosali cohorts (Table 3).

**Table 2:** **Baseline determinants of persistent knee pain in men, univariate analyses of Gazel and Cosali cohorts**

	Gazel men, N = 3190				Cosali men, N = 376			
	#	OR (95% CI)	P	P	#	OR (95% CI)	P	P
**Age at baseline, in years**	.		.	0.6876	.		.	0.4514
20-39	0	-	-		159	1.00 (.-.)	.	.
40-49	0	-	-		132	1.00 (0.63-1.59)	0.9903	.
50-59	709	1.00 (.-.)	.	.	85	1.37 (0.81-2.34)	0.2441	.
60-64	1657	1.00 (0.84-1.20)	0.963	.	0	-	-	.
65-69	824	0.93 (0.76-1.15)	0.514	.	0	-	-	.
**BMI at baseline**	.		.	**<.0001**	.		.	**0.0428**
Normal weight, missing	1074	1.00 (.-.)	.	.	191	1.00 (.-.)	.	.
Overweight	1597	**1.34 (1.14-1.57)**	**0.0003**	.	146	**1.72 (1.11-2.66)**	**0.0155**	.
Obesity	519	**1.67 (1.34-2.08)**	**<.0001**	.	39	1.58 (0.79-3.18)	0.1994	.
**Socio-professional category at 35 years/at baseline**	.		.	0.1694	.		.	**0.0107**
Executives, others, missing	541	1.00 (.-.)	.	.	33	1.00 (.-.)	.	.
Associate professionals or technicians	1834	1.08 (0.89-1.32)	0.4232	.	99	**3.20 (1.35-7.58)**	**0.0082**	.
Employees	160	0.89 (0.62-1.27)	0.5227	.	26	**6.00 (1.94-18.60)**	**0.0019**	.
Manual workers	655	1.24 (0.98-1.57)	0.074	.	218	**3.39 (1.51-7.63)**	**0.0032**	.
Carrying/Handling heavy loads**	.		.	**<.0001**	.		.	0.0928
None, missing	1236	1.00 (.-.)	.	.	201	1.00 (.-.)	.	.
Short or moderate exposure	938	1.09 (0.92-1.30)	0.3201	.	86	1.61 (0.96-2.69)	0.0699	.
Long or severe exposure	1016	**1.51 (1.27-1.80)**	**<.0001**	.	89	1.55 (0.93-2.57)	0.0906	.
Carrying/Handling heavy loads**	.		.	**0.0007**	.		.	**0.0295**
No, missing	1236	1.00 (.-.)	.	.	201	1.00 (.-.)	.	.
Yes	1954	**1.29 (1.11-1.49)**	**0.0007**	.	175	**1.58 (1.05-2.38)**	**0.0295**	.
**Kneeling**	.		.	**<.0001**	.		.	0.1040
None, missing	1441	1.00 (.-.)	.	.	129	1.00 (.-.)	.	.
Short or moderate exposure	970	**1.22 (1.03-1.44)**	**0.0202**	.	132	1.51 (0.93-2.47)	0.0963	.
Long or severe exposure	779	**1.62 (1.35-1.95)**	**<.0001**	.	115	**1.66 (1.00-2.76)**	**0.0494**	.
**Kneeling**	.		.	**<.0001**	.		.	**0.036**
No, missing	1441	1.00 (.-.)	.	.	129	1.00 (.-.)	.	.
Yes	1749	**1.38 (1.19-1.59)**	**<.0001**	.	247	**1.58 (1.03-2.43)**	**0.036**	.
**Walking up more than 10 flights of stairs**	.		.	0.3432				
None, missing	2363	1.00 (.-.)	.	.				
Short exposure	551	1.15 (0.95-1.39)	0.1561	.				
Long exposure	276	1.07 (0.83-1.39)	0.5837	.				
**Sick leave for depression**	.		.	0.1616				
No	2832	1.00 (.-.)	.	.				
Yes	358	1.18 (0.94-1.48)	0.1616	.				
**Pain intensity**	.		.	**<.0001**	.		.	**0.0004**
Low, missing	1741	1.00 (.-.)	.	.	106	1.00 (.-.)	.	.
High	1449	**1.46 (1.26-1.69)**	**<.0001**	.	95	**2.06 (1.15-3.69)**	**0.0154**	.
No presentpain					175	0.71 (0.44-1.15)	0.1612	.
**Past history of knee injury**	.		.	**<.0001**				
No, missing	1922	1.00 (.-.)	.	.				
Yes	1268	**1.50 (1.29-1.74)**	**<.0001**	.				
**Self-assessed origin of knee pain**	.		.	**<.0001**				
Degenerative osteoarthritis	1349	**1.91 (1.64-2.23)**	**<.0001**	.				
Other	1559	1.00 (.-.)	.	.				
Not completed	282	0.80 (0.62-1.03)	0.0871	.				
**Pain in other areas at baseline**	.		.	**0.0001**	.		.	0.7185
No pain	380	1.00 (.-.)	.	.	33	1.00 (.-.)	.	.
Hip pain	740	**1.69 (1.31-2.18)**	**<.0001**	.	110	1.37 (0.63-2.99)	0.4262	.
Pain in other areas	2070	**1.27 (1.02-1.59)**	**0.0321**	.	233	1.23 (0.59-2.55)	0.5785	.

**Gazel: Carrying loads of more than 10 kg, Cosali: Handling loads of more than 4 kg.

In bold: Significant associations (p < 0.05).

**Table 3:** **Baseline determinants of persistent knee pain in women, univariate analyses of Gazel and Cosali cohorts**

	Gazel women, N = 950				Cosali women, N = 261			
	#	OR (95% CI)	P	P	#	OR (95% CI)	P	P
**Age at baseline, in years**				0.8944				0.1397
20-39	0	-	-		98	1.00 (.-.)		
40-49	0	-	-		91	1.77 (1.00-3.16)	0.0517	
50-59	324	1.00 (.-.)			72	1.47 (0.80-2.71)	0.2176	
60-64	420	0.93 (0.68-1.26)	0.638	.	0	-	-	
65-69	206	0.95 (0.66-1.38)	0.7949	.	0	-	-	
**BMI at baseline**	.			**0.0179**	.		.	0.4198
Normal weight, missing	485	1.00 (.-.)			159	1.00 (.-.)	.	.
Overweight	317	**1.40 (1.04-1.90)**	**0.028**		74	1.12 (0.64-1.94)	0.6873	.
Obesity	148	**1.62 (1.08-2.44)**	**0.0198**		28	1.73 (0.76-3.93)	0.1896	
**Socio-professional category at 35 years/at baseline**				0.4887				0.911
Executives, others, missing	111	1.00 (.-.)			12	1.00 (.-.)		
Associates professionals or technicians	504	1.24 (0.81-1.90)	0.3314		44	1.28 (0.35-4.65)	0.7094	
Employees	330	1.16 (0.74-1.81)	0.5269		128	1.36 (0.41-4.50)	0.6178	
Manual workers	5	0.39 (0.06-2.44)	0.3145		77	1.51 (0.44-5.19)	0.5096	
Carrying/Handling heavy loads**				0.4154				0.4789
None, missing	822	1.00 (.-.)			187	1.00 (.-.)		
Short or moderate exposure	99	1.04 (0.66-1.62)	0.8782		47	1.39 (0.73-2.65)	0.3123	
Long or severe exposure	29	0.61 (0.29-1.28)	0.1921		27	1.41 (0.62-3.17)	0.4103	
Carrying/Handling heavy loads**				0.6448				0.225
No, missing	822	1.00 (.-.)			187	1.00 (.-.)		
Yes	128	0.91 (0.62-1.35)	0.6448		74	1.40 (0.81-2.40)	0.225	
**Kneeling**				0.8991				0.8211
None, missing	901	1.00 (.-.)			106	1.00 (.-.)		
Short or moderate exposure	40	1.18 (0.59-2.35)	0.6446		96	0.85 (0.49-1.48)	0.564	
Long or severe exposure	9	1.01 (0.25-4.06)	0.9907		59	1.00 (0.53-1.88)	0.9906	
**Kneeling**				0.6746				0.6851
No, missing	901	1.00 (.-.)			106	1.00 (.-.)		
Yes	49	1.14 (0.61-2.13)	0.6746		155	0.90 (0.55-1.48)	0.6851	
Carrying/Handling heavy loads * Kneeling				0.4537				0.785
<= short or moderate exposure, missing	915	1.00 (.-.)			187	1.00 (.-.)		
One long or severe exposure	32	0.63 (0.31-1.29)	0.2087	.	62	1.23 (0.69-2.18)	0.4873	
Both long or severe exposure	3	0.99 (0.09-10.91)	0.9904	.	12	1.08 (0.34-3.46)	0.9000	
**Walking up more than 10 flights of stairs**				0.4544				
None, missing	922	1.00 (.-.)						
Short exposure	20	1.50 (0.54-4.18)	0.4331					
Long exposure	8	0.50 (0.12-2.02)	0.3316					
**Sick leave for depression**				0.7561				
No	576	1.00 (.-.)						
Yes	374	0.96 (0.73-1.26)	0.7561					
**Pain intensity**				**<.0001**	.		.	**0.0057**
Low, missing	515	1.00 (.-.)			61	1.00 (.-.)	.	.
High	435	**1.91 (1.44-2.52)**	**<.0001**		64	**2.58 (1.25-5.33)**	**0.0106**	.
No presentpain					136	0.96 (0.53-1.77)	0.9083	.
**Past history of knee injuries**				0.0655				
No, missing	652	1.00 (.-.)	.					
Yes	298	1.32 (0.98-1.78)	0.0655					
**Self-assessed origin of knee pain**				**0.0091**				
Degenerative osteoarthritis	543	**1.43 (1.06-1.92)**	**0.0183**	.				
Other	305	1.00 (.-.)	.	.				
Not completed	102	0.82 (0.52-1.29)	0.3909	.				
**Pain in other areas at baseline**				**0.0012**				0.1205
No pain	69	1.00 (.-.)	.		8	1.00 (.-.)		
Hip pain	345	**2.67 (1.58-4.52)**	**0.0003**		107	0.77 (0.17-3.37)	0.7243	
Pain in other areas	536	**2.14 (1.29-3.55)**	**0.0032**		146	0.47 (0.11-2.03)	0.3112	

**Gazel: Carrying loads of more than 10 kg, Cosali: Handling loads of more than 4 kg.

In bold: Significant associations (p < 0.05)

The first model adjusted on age, BMI and carrying/handling heavy loads and kneeling yielded results similar to the model also adjusted on indicators of severity (Table 4). The following indicators of severity of KP at baseline were significant in the multivariate analyses: greater intensity of KP at baseline, past history of knee injury in men, degenerative osteoarthritis as self-assessed origin of pain (available in Gazel only) and pain in other areas (significant in Gazel, not in Cosali). The persistence of KP in men was associated with exposure to kneeling in multivariate analyses adjusted on indicators of severity of baseline KP in Gazel and Cosali cohorts. A significant interaction between intensity of KP at baseline and kneeling was found in Gazel cohort in women (p = 0.0143) and in Cosali cohort in men (p = 0.0536). The OR between the persistence of KP at follow up and kneeling was of 4.66 (1.02-21.35) among women in Gazel with high intensity of KP at baseline and of 4.35 (1.27-14.91) among men in Cosali with high intensity of KP at baseline.

**Table 4:** **Occupational exposure as risk factors for persistent knee pain, multivariate analyses of Gazel and Cosali cohorts**

	Men				Women			
	Gazel, N = 3190		Cosali, N = 376		Gazel, N = 950		Cosali, N = 261	
	OR (95% CI)	P	OR (95% CI)	P	OR (95% CI)	P	OR (95% CI)	P
Model 1*								
Carrying/Handling heavy loads***		0.6212		0.0942		0.3628		0.1259
No, missing	1.00 (.-.)		1.00 (.-.)		1.00 (.-.)		1.00 (.-.)	
Yes	1.05 (0.86-1.28)		1.45 (0.94-2.23)		0.83 (0.55-1.25)		1.56 (0.88-2.76)	
**Kneeling**		**0.0067**		**0.0424**		0.4817		0.6423
No, missing	1.00 (.-.)		1.00 (.-.)		1.00 (.-.)		1.00 (.-.)	
Yes	**1.31 (1.08-1.60)**		**1.62 (1.02-2.58)**		1.26 (0.66-2.43)		0.88 (0.52-1.49)	
Model 2**								
Carrying/Handling heavy loads***		0.8665		0.0706		0.3163		0.1166
No, missing	1.00 (.-.)		1.00 (.-.)		1.00 (.-.)		1.00 (.-.)	
Yes	1.02 (0.83-1.25)		1.51 (0.97-2.38)		0.81 (0.53-1.23)		1.61 (0.89-2.91)	
**Kneeling**		**0.0185**		**0.0496**		0.7488		0.8053
No, missing	1.00 (.-.)		1.00 (.-.)		1.00 (.-.)		1.00 (.-.)	
Yes	**1.27 (1.04-1.56)**		**1.62 (1.00-2.63)**		1.12 (0.57-2.18)		0.93 (0.54-1.60)	

*Model adjusted on age and BMI.

**Model adjusted on age, BMI , intensity of pain, pain in other areas, self-assessed origin of KP (Gazel only), past history of knee injury (Gazel only).

***Gazel: Carrying loads of more than 10 kg, Cosali: Handling loads of more than 4 kg.

In bold: Significant associations (p < 0.05).

## Discussion

In this study, high levels of episodes of KP in retirees and working populations were demonstrated in both men and women, with only 33% to 50% of recovery from KP two to nine years after the initial episode of KP.

The definitions of persistence of KP are different in the literature, and involve pain intensity [3], the perceived global evolution of pain [2], and duration of pain [5–7]. These different definitions could explain the wide range of recovery rates reported in the literature and this is highlighted in this study by the fact that the natural history and the duration of KP at follow-up depended considerably on the duration of KP at baseline. Most studies are based on two measures, with a one-year period between them [2, 5–7], in contrast to our study that was based on a long period of follow-up. The recovery rate was estimated for a one-year period in order to have a comparable measure between the two cohorts and with the literature. These annual recovery rates varied between 16 and 19% for men and 12 and 18% for women. They were low in the range of recovery rates previously reported and this could be explained either by the calculation methods used or by the less restrictive definition of KP applied. The definition was frequently not very precise, whereas a specific pain index for knee osteoarthritis, for example, provide more details on the type and period of pain [17]. An episode during the previous year may have been forgotten, particularly in case of short duration. Moreover, due to the small numbers in some categories (especially for the Cosali cohort), the definition was wide, comprising all types of KP, including those with longstanding and severe pain and those with pain of short duration.

It was expected that BMI, the intensity of KP, degenerative osteoarthritis, pain in other areas at baseline and a past history of knee injury would be found to be associated with the persistence of KP in men and women [2, 6]. Age was not found to be associated with persistent KP, in contrast to previous studies [2, 5, 6]. However, older age in the Gazel study, with a higher proportion of long-lasting pain than the Cosali study, was consistent with the known relationship between age and KP.

Occupational factors appeared to be associated with the persistence of KP in men in the Gazel cohort, i.e. by definition at least 6 years after exposure and during retirement. In particular, kneeling and handling/carrying loads were found to be associated with significant dose–response effects. Even after adjustment on the severity of KP, exposure to kneeling (versus no exposure) was significantly associated with the persistence of KP in men. Climbing stairs was not found to be associated with the persistence of KP, in contrast to previous studies on knee osteoarthritis [18]. The Gazel cohort members were not exposed to occupational risk factors during the follow-up period. Although the mechanism could not be explored here, we hypothesize that the effect of previous occupational exposure was due to the severity of osteoarthritis and delayed severe symptoms, bearing in mind the relationship between occupational exposure and osteoarthritis and the discordance between clinical and radiographic evidence of knee osteoarthritis at the beginning of the disease [19–21]. The risks of persistence of KP according to occupational exposure (all durations or intensity taken together) were similar in the Cosali cohort and in the Gazel cohort. In the Cosali cohort, the socio-professional category of men still in employment was significant, with lower risk of persistence for executives. As current occupational exposure was related to past exposure, the indirect mechanism mediated by the baseline severity of KP could also be hypothesized in the Cosali cohort. Moreover, occupational exposure seemed to be maintained during the follow-up period for a large proportion of the Cosali cohort members and this could have had a negative effect on recovery, due to inadequate resting time or awkward postures in the job. The measurement of occupational exposure in the two cohorts had advantages and disadvantages: in the Gazel study, the retrospective measurement could have been affected by a bias of memory (that may have overestimated the associations) but did provide a global view of exposure during a whole career; in the Cosali study, the current measurement of exposure was incomplete because it did not elicit the duration of past exposure but this prospective measurement is less prone to bias of memory. Using self-reported measurements is a limitation. However, a review in 2005 highlighted that self-reported answers to questions concerning physical work demands showed good reproducibility [22].

The results observed for occupational exposure for men in these cohorts were not found for women. Overall, levels of occupational exposure were lower for women (3% to 13% exposed in the Gazel study) and the analyses might have suffered from lack of power in the Cosali study. This result has already been reported for female healthcare workers [6] but further studies with more power will be necessary to confirm or disprove these results.

One important limitation in this study was loss to follow-up. In the Gazel cohort, although the missing questionnaires may have been due to health problems [11], the rate of loss to follow-up was very low and the persistence rates estimated were robust to extreme scenarios of loss to follow-up. In the Cosali cohort, those lost to follow-up may have had unstable professional situations or health problems and the percentage of recovery may have varied by as much as 100%. However, the study on the Cosali cohort was crucial because it provided the opportunity to assess the direct effects of maintaining exposure on the persistence of KP. Moreover the occupations represented were diverse and representative of the regional workforce.

## Conclusions

This study highlights the role of occupational factors in the persistence of KP, in particular kneeling and handling/carrying loads for men. Such occupational exposure should as far as possible be reduced for workers to prevent the persistence of KP, particularly among workers with high intensity of pain.

## Authors’ original submitted files for images

Below are the links to the authors’ original submitted files for images. Authors’ original file for figure 1 Authors’ original file for figure 2
