# Supplementary figures and images for: Long-term persistence of knee pain and occupational exposure in two large prospective cohorts of workers
Source: BMC Musculoskelet Disord. 2014 Dec 5;15:411. doi: 10.1186/1471-2474-15-411 (PMC4289228; doi:10.1186/1471-2474-15-411)

## Annual auto-questionnaires

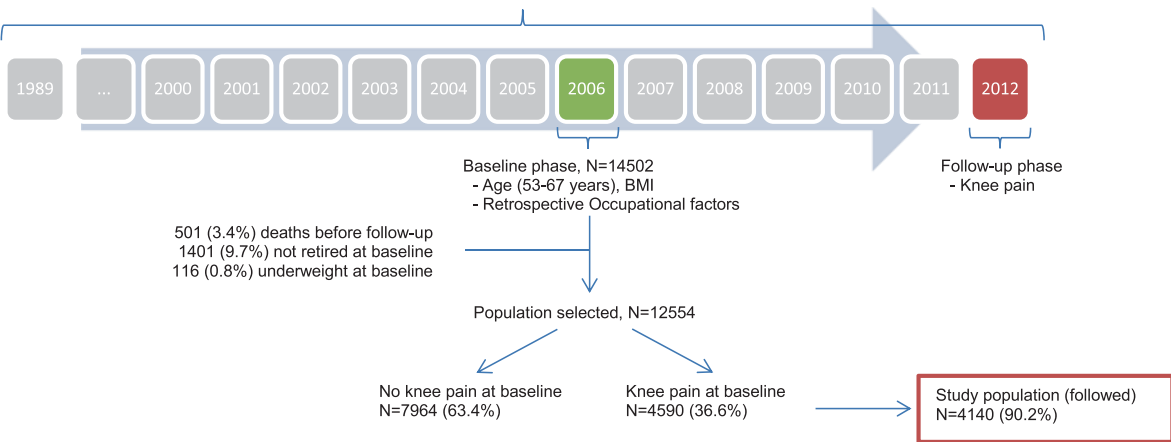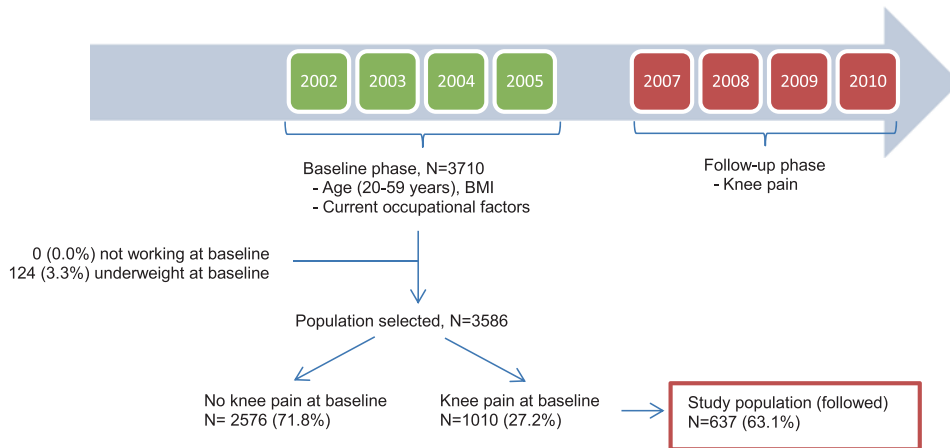

Supplement: Supplementary file 1 — Authors’ original file for figure 1 [file 12891_2014_2348_MOESM1_ESM.pdf]

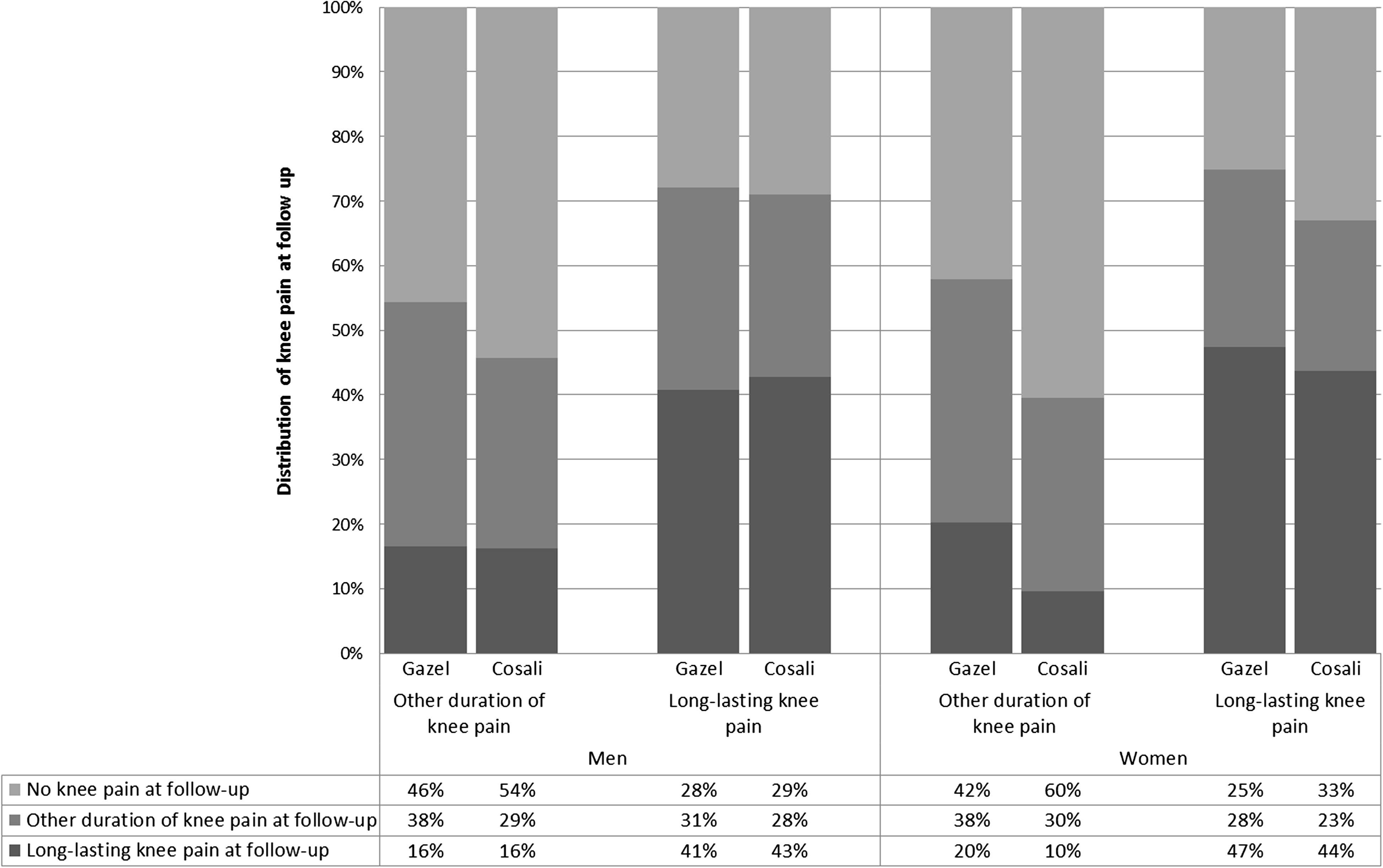

Supplement: Supplementary file 2 — Authors’ original file for figure 2 [file 12891_2014_2348_MOESM2_ESM.tif]
